# MicroRNAs Expression in Triple Negative vs Non Triple Negative Breast Cancer in Tunisia: Interaction with Clinical Outcome

**DOI:** 10.1371/journal.pone.0111877

**Published:** 2014-11-04

**Authors:** Imen Medimegh, Ines Omrane, Maud Privat, Nancy Uhrhummer, Hajer Ayari, Fadoua Belaiba, Farhat Benayed, Khaled Benromdhan, Sylvie Mader, Ives-Jean Bignon, Amel Benammar Elgaaied

**Affiliations:** 1 Laboratory of Genetics, Immunology and Human Pathology, Faculty of Sciences of Tunis, University of Tunis El Manar, Tunis, Tunisia; 2 Salah Azaiez Institute of Carcinology, Tunis, Tunisia; 3 Laboratory of Genetics and Molecular Diagnostic, Centre Jean Perrin, Clermont-Ferrand, France; 4 Laboratory of Molecular Screening in the Treatment of Breast Cancer, Immunology and Cancer Research Institute, University of Montreal, Montreal, Canada; Rutgers - New Jersey Medical School, United States of America

## Abstract

**Introduction:**

MicroRNAs are small, non coding regulatory molecules containing approximately 21 to 25 nucleotides. They function as controllers of expression at post transcriptional levels of most human protein-coding genes and play an essential role in cell signaling pathways. The objective of the present study is to evaluate the expression profile of the following micro-RNAs: miR-10b, miR-17, miR-21, miR-34a, miR-146a, miR-148a and miR-182, and to determine their possible interaction in triple-negative and non triple-negative primary breast cancers based on clinical outcome.

**Methods:**

60 triple-negative and non triple-negative breast cancer cases, along with their corresponding normal samples were investigated in relation to the expression of the seven studied miRNAs using qPCR Syber Green.

**Results:**

We observed that miR-21, miR-146a and miR-182 were significantly over expressed in triple negative breast cancer. Moreover, miR-10b, miR-21 and miR-182 were significantly associated to lymph node metastases occurrence in triple negative breast carcinoma while only miR-10b was associated with grade III in non triple negative breast cancer cases. Almost all the analyzed microRNAs were strongly associated with patients’ genico-obstetric history in non triple negative breast cancer cases except for miR-34a. All the studied microRNAs were strongly correlated with the use of the contraceptive pills in non triple negative breast cancer groups. The additive effect of hormonal factors in triple negative breast cancer cases showed an association with all the studied miRs except for miR-34 and miR-146a.

**Conclusion:**

The studied microRNAs are strongly influenced by environmental factors especially with hormonal patients’ history. Moreover, miR-10b, miR-21 and miR-182 could be defined as biomarkers in breast cancer to predict both lymph node metastases and grade III occurrence.

## Introduction

Breast cancer is the most frequent neoplasm affecting women worldwide. The development of high-throughput array-based gene expression profiling platforms allows the classification of primary breast cancer according to three major subtypes: luminal (A and B), basal-like and Her2/neu-overexpressing [1].

The expression of the estrogen receptor (ER), progesterone receptor (PR) and the human epidermal growth factor receptor (Her2/neu), usually determines the proper therapeutic response and general disease prognosis [2]. Breast tumors expressing the hormonal receptors are usually luminal A and B breast cancer subtypes which are characterized as a favorable prognosis since they are followed up with hormonal anti-tumor treatments, such as aromatase inhibitors and tamoxifen, which target the estrogen receptor [3]. Tumors with over expressed HER-2 are targeted by trastuzimab, which blocks the human epidermal growth factor receptor [4]. However, triple negative breast cancer expresses none of the three breast cancer markers (ER, PR and Her2). This phenotype is associated with a poorer prognosis with a high risk of relapse since it is treated with conventional and moderately successful chemotherapies [5]. Recent studies show a relative efficiency of *PARP* inhibitors, anti- *EGFR* and tyrosine kinase inhibitors [6], [7], [8]. There is often a resistance which is associated with these treatments [9]. Triple-negative breast cancer profile is classified as normal breast-like if basal cytokeratins and the epidermal growth factor receptor are lacking, and as basal-cell-like cancers when basal cytokeratines (cytokeratin 5 and 6 and/or cytokeratin 14) are expressed [10]. Triple negative tumors represent 8–15% of all breast cancer cases and 10–15% of basal-like tumors [11]. Triple-negative and basal-like breast cancers are often associated with high proliferation, high grade, young age (<38 years), lack of *BRCA1* protein expression and aggressive clinical behavior (lymph node and distant metastases occurrence) [12].

Several studies classified the triple negative breast carcinoma subtype according to epigenetic profiles that are modulated by the micro-environment of the tumor cell [13]. Recently an extra level of gene regulation involved in the cell signaling pathways in both normal and tumor tissues manifested in small non-coding RNA molecules or microRNAs (miRs). They are small, regulatory molecules, approximately 20–25 nucleotides in length belonging to the largest family of non-coding RNAs [14]. They have an important role in gene silencing by binding directly and specifically to mRNA molecules and inducing their degradation (perfect match) or a decrease in the rate of the protein translation (imperfect match) [15]. Some microRNAs are down-regulated in a number of different tumors and the re-introduction of these microRNAs has been shown to impair the viability of cancer cells, because of their function as tumor-suppressor genes with an anti-proliferative and pro-apoptotic activity role. They are under-expressed in cancer cells. In contrast to the tumor-suppressor microRNAs, the oncogenic miRNAs (oncomiRs) display an anti-apoptotic activity and are over-expressed in cancer cells [16]. Therefore, alterations in the expression of these miRNAs, could stimulate tumorigenesis [16]. Several studies have demonstrated that different types of cancer at different developmental stages display unique expression profiles of different microRNAs [17]. This suggests the use of these unique microRNAs expression patterns as tumor diagnostic and prognostic biomarkers, but also for future microRNA gene therapy.

In the present study, we explored the expression levels of seven microRNAs: miR-10b, miR-17, miR-21, miR-34a, miR-146a, miR-148a and miR-182 in both triple negative (TNBC) and non triple negative breast carcinoma (NTNBC). Moreover, we investigated their possible interactions with the clinical outcome along with genico-obstetric history of patients.

## Materials and Methods

### Patients

Two groups of patients are tested in this study, the first with a triple negative breast cancer profile and the second with a non triple negative breast cancer profile (Luminal A, Luminal B, and HER-2). Formalin-fixed, paraffin-embedded (FFPE) tissue files were drawn from the department of pathological anatomy of Salah Azaiez Institute of Tunis.

We managed to recover 30 FFPE tissues for each group of patients with their corresponding normal tissues. Three 10 mm cores were obtained from both the tumor and the normal tissue. Essential information includes stage, grade, lymph nodes metastasis and distant metastasis along with data on ER, PR and HER-2 tumor expression and the genico-obstetric history of patients were collected from the Salah Azaiez Institute data base. For tumor tissue sampling, a great effort was made to avoid any adjacent normal tissue and to isolate areas of tissue containing >70% of tumor cells. All personal data were blinded to guarantee patients’ protection. The procedures were in agreement with the regulations for use of human material in research issued by the Medical Ethics Committee of Pasteur Institute of Tunis. Written informed consent was given by all participants for their clinical data and tissue samples to be used in this study and was approved by Dr M. Samir BOUBAKER president of the Medical Ethics Committee of Pasteur Institute of Tunis.

### Quantitative real time PCR

Total RNA was extracted by the miRNeasy FFPE Kit (QIAGEN, France) according to the manufacturer’s protocol. The concentration and quality of the extracted RNA was measured by a NanoDrop ND-2000c spectrophotometer. The reverse transcription and c.DNA synthesis from isolated total RNA was performed with the miScript Reverse Transcription Kit (QIAGEN, France). RNA samples were transcribed to c.DNA by manufacturer optimized oligo-dT and random primers. c.DNA synthesis was performed at 37°C for 60 min, and the reaction was inactivated by incubation at 95°C for 5 min. The oligo-dT primers were identified through a universal tag sequence on the 5′ end, which allows amplification in the real-time PCR step. The analyzed miRNAs are involved in pathways dealing with tumorigenesis, invasion, angiogenesis, breast hormone regulation, lymph node and distant metastasis (Table 1). We chose potentially prognostic miRNAs that could be in relation with the histopathological information (tumor grade and stage), prediction of metastasis or risk of relapse. Selected microRNA primers were obtained from miScript Primer Assay (QIAGEN, France), as shown in Table 2.

**Table 1:** Tumorigenesis and targeted genes of the selected microRNAs in breast cancer and other human malignancies.

microRNA	Tumorigenesis	Role and targetedgenes	References
miR-10b	Breast cancer	Involved in invasion andcell motility Targets the 3′UTRof *HOXD10*. Upregulated by*Twist* and *Snai1*	[31]
miR-17	Breast cancer	Has an anti-apoptic role.Targets *BRCA1* and *P53* genes	[28], [32]
miR-21	Leukemia, lymphoma,breast, ovarian,cervical, colorectaland prostate cancer.	Plays a key role in resistingprogrammed cell death in cancercells. Invasion and metastasis,genome instability. It targets *P53*and other tumor suppressor genes	[33], [34], [35]
miR-34a	Breast cancer(Luminal A), prostate,pancreaticcancer	Targets 3′UTR of *MAGE-A*that down regulates *P53*and *Bcl-2*.Plays a role as tumor suppressorgene in human pancreatic cancerwith loss of *p53*	[28]
miR-146a	Breast andcolorectal cancer	Downregulates *BRCA1* (3′UTR)	[40]
miR-148a	Several cancer types	Downregulates *C-MYC0, CDK6*and *TGIF2*. It has been found to bedownregulated in several tumortypes and has been suggested tobe a tumor suppressor gene	[28]
miR-182	Breast and ovariancancer	Targets the 3′UTR of *BRCA1*.Downregulates *PFN1*. Involvedin the resistance of *PARP* inhibitors	[41]

**Table 2:** List of the analyzed microRNAs.

Assay name	QIAGEN Cat No	miR-BaseAccession No	Mature miRNA sequence
Hs_miR-10b_3	MS00031269	MIMAT0000254	UACCCUGUAGAACCGAAUUUGUG
Hs_miR-17_2	MS00029274	MIMAT0000070	CAAAGUGCUUACAGUGCAGGUAG
Hs_miR-21_2	MS00009079	MIMAT0000076	UAGCUUAUCAGACUGAUGUUGA
Hs_miR-34a_1	MS00003318	MIMAT0000255	UGGCAGUGUCUUAGCUGGUUGU
Hs_miR-146a_1	MS00003535	MIMAT0000449	UGAGAACUGAAUUCCAUGGGUU
Hs_miR-148a_1	MS00003556	MIMAT0004549	UCAGUGCACUACAGAACUUUGU
Hs_miR-182_2	MS00008855	MIMAT0000259	UUUGGCAAUGGUAGAACUCACACU

Expression of miRNAs was measured using the miScript SYBR Green PCR Kit and miScript Primer Assays (Qiagen, France) according to the manufacturer’s protocol. All amplification reactions were performed in triplicates. One of the relative standards was repeatedly used as inter-assay calibrator to normalize different PCR efficiencies among runs. For each sample, the standard control used is RNU6B-6 as endogenous control. RNU6B-6 corresponds to a small nucleolar RNA that was selected by a previous study from the best performing controls for miR quantification assays. Its mean expression level was used as a reference. miRNA expression levels (2^−ΔCt^) were normalized to the reference and to the mean expression of the normal samples using the ΔΔCt method. Data were standardized by log2 transformation.

### Statistical analysis

Normality of the data distribution was checked by Kolmogorov-Smirnov test. Differences in the expression levels between breast tumor and normal tissue were measured using the Mann-Whitney U test. The association between miRNA expression and clinical features were analyzed via the Spearman’s correlation test. Numerical values are expressed as the mean ± standard deviation (SD). Statistical significance was set at the 95% level (p<0.05). All statistical analyses were performed with SPSS 20 (SPSS, IBM) software.

## Results

### Clinical outcome

The subjects of our study consisted in 60 patients with primary breast carcinoma. These cases were divided into two groups: 30 patients with triple negative breast carcinoma and 30 patients with non triple negative breast cancer profile including 16 cases with the Luminal A subtype, 10 with the Luminal B subtype and 4 with the HER-2 subtype. Data concerning the expression of the three markers (ER, PR and HER-2) by routine immunohistochemistry along with clinical and personal information were collected from the archive of the Salah Azaiez Institute data base. We first compared the two studied groups in terms of the clinical data including age at diagnosis, histological breast cancer type, lymph node metastases, distant metastases, grade and stage, along with genico-obstetric information including age of first menstruation, age at first pregnancy, number of completed pregnancy, breastfeeding, contraceptive pills and body mass index (Table 3). We did not consider the subtypes of non triple negative breast cancer (Luminal A, Luminal B and HER-2) because they are presented by a small number of subjects. We did not record any significant difference in the analyzed data between the two groups except for the age at diagnosis and grade of the tumor. We observed that young women under the age of 38 at diagnosis represent 40% in the triple negative breast cancer group while they are only 13.4% in the non triple negative breast cancer group, *P* value = 0.04 and OR (4.33–1.2). We also noticed that grade III concerns 90% of the patients with triple negative breast cancer; however, it is presented in only 63.3% of the patients with non triple negative breast cancer; *P* value = 0.03 and OR (5.2–1.2) (Table3).

**Table 3:** Comparison of the clinico-pathological features along with patients’ genico-obstetric history among triple negative and non triple negative mammary tumors.

Clinical features		Triple negativebreast cancer		Non triple negativebreast cancer		Corrected*P* value (OR)
		N = 30	(%)	N = 30	(%)	
**^1^Age at** **diagnoses**	**<38 years**	12	(40)	04	(13.	**0.04** (4.3–1.2)
	**>38 years**	18	(60)	26	(86.6)	
**^2^Histological** **type**	**CCI**	27	(90)	26	(86.6)	1
	**CLI**	02	(6.7)	02	(6.7)	
	**CCIS**	0	(0)	02	(6.7)	
	**CS**	01	(3.3)	0	(0)	
**^3^Lymph node metastases**	**No**	20	(66.6)	24	(80)	0.3
	**Yes**	10	(33.4)	06	(20)	
**^4^Grade**	**I**	0	(0)	02	(6.	**0.03** (5.2–1.2)
	**II**	03	(10)	09	(30)	
	**III**	27	(90)	19	(63.3)	
**^5^Distance** **metastasis**	**No**	23	(76.6)	26	(86.	0.5
	**Yes**	07	(23.4)	04	(13.4)	
**^6^Stade**	**T0**	0	(0)	02	(6.7)	0.3
	**T1–T2**	19	(63.4)	23	(76.6)	
	**T3–T4**	11	(36.6)	05	(16.7)	
**^7^Age of first menstruation**	**<13 years**	11	(36.7)	09	(30)	0.8
	**>13 years**	13	(43.3)	14	(46.7)	
	**Unknown**	06	(20)	07	(23.3)	
**^8^Age of first pregnancy**	**No pregnancy**	06	(20)	10	(33.4)	0.8
	**<25 years**	10	(33.4)	08	(26.6)	
	**>25 years**	09	(30)	09	(30)	
	**Unknown**	05	(16.6)	03	(10)	
**^9^Number of** **completed pregnancy**	**0**	10	(33.4)	14	(46.7)	0.2
	**one at least**	15	(50)	09	(30)	
	**Unknown**	05	(16.6)	07	(23.3)	
**^10^Breastfeeding**	**No**	14	(46.7)	14	(46.7)	0.9
	**Yes**	12	(40)	10	(33.4)	
	**Unknown**	04	(13.3)	06	(20)	
**^11^Contraceptive pills**	**No**	16	(53.4)	19	(63.4)	0.4
	**Yes**	10	(33.4)	06	(20)	
	**Unknown**	04	(13.3)	05	(16.6)	
**^12^Body mass** **index**	**<30**	13	(43.3)	09	(30)	0.1
	**>30**	10	(33.4)	18	(60)	
	**Unknown**	07	(23.3)	03	(10)	

*P* in blot <0.05 is significant. CI = 95%; 2- Compared between (CCI and CLI+CCIS+CS); 4-Compared between (III and I+II); 6- Compared between (T1–T2 and T0+T3–T4); 7- Compared between <12 years and >12 years; 8- Compared between <25 years and >25 years; 9 -Compared between 0 and one at least; 10- Compared between No and Yes; 11- Compared between No and Yes; 12- Compared between <30 and >30.

### miRNA expression levels in breast cancer versus normal tissue

We analyzed the expression of seven microRNAs (miR-10b; miR-21; miR-34a; miR-146a; miR-148a and miR-182) among the two groups of breast cancer types in both tumor and normal adjacent tissues. The choice of the seven studied miRs was determined according to the literature and to their potential involvement in breast cancer in general and in mammary tumor subtypes in particular (Table 1). We analyzed the seven selected microRNAs for the first time in the Tunisian population, knowing that micro-RNAs are influenced by several factors such as environmental and hormonal factors. The expression levels were normalized with the corresponding mean value of the reference gene *RNU6B*, the samples were checked for outliers to be excluded. The normalized expression data of the seven analyzed miRNAs were log2 transformed. We first compared the expression levels of the analyzed microRNAs between tumor and normal tissues in both triple negative and non triple negative breast cancer groups (mean ± SD). The relative expression ratio *R* which is presented as the n-fold change in gene expression, allowed comparing the overall level of each miRNA. The value of *R*>1 represented miRNAs over expression in both triple negative and non triple negative mammary tumors relative to paired normal tissue (Table 4). All the analyzed microRNAs across the two tested groups are upregulated in tumors compared to normal tissue and exhibited higher than two-fold differential expression with *P* value <0.05 except for miR-10b in TNBC and NTNBC with *R* ratio = 1.12 and 0.9 respectively and *P* value = 0.27 and 0.33 respectively. The highest difference between tumors and normal tissues concerns miR-146a in triple negative breast cancer group with *R* ratio = 9.7 and *P* value <0.01 (Table 4).

**Table 4:** Comparison of miRs expression levels in normal and tumor mammary tissues among triple negative and non triple negative mammary tumors.

micro RNA	Triple negative mammary tumors N = 30				Non triple negative mammary tumors N = 30			
	Tumor tissueCT value(mean ± SD)	Normal tissueCT value(mean ± SD)	Expressionratio *R*	*P* value	Tumor tissueCT value(mean ± SD)	Normal tissueCT value(mean ± SD)	Expressionratio *R*	*P* value
**miR-10a**	9.3379±1.93	10.0097±2.84	1.12	0.27	9.6662±1.8	10.1462±2.69	0.9	0.33
**miR-17**	5.5598±1.9	8.5298±3.02	4.38	**<0.01**	7.577±1.66	8.8785±1.98	4.61	**<0.01**
**miR-21**	0.9867±2.25	4.4226±3.31	4.56	**<0.01**	3.4225±1.47	4.6714±1.68	3.37	**0.02**
**miR-34**	9.8267±1.77	11.9091±1.95	5.04	**<0.01**	7.0984±2	10.3808±2.46	6.32	**<0.01**
**miR-146a**	3.5776±1.95	7.4624±1.57	9.7	**<0.01**	6.5827±1.72	8.9785±2.87	3.93	**<0.01**
**miR-148a**	5.7550±1.7	8.0905±3.32	3.48	**0.002**	4.6166±1.83	6.5744±1.76	6.11	**<0.01**
**miR-182**	8.1276±2	12.9789±2.63	7.27	**<0.01**	10.3669±1.64	12.1073±1.77	6.31	**<0.01**

Blot letters indicate *p* Value <0.01, SD: standard deviation.

### Comparison of miR expression in TNBC versus NTNBC

We assessed the mean fold expression levels (mean ± SD), that was Log N transformed, of the different studied microRNA between both triple negative and non triple negative breast cancer cases after normalization with the expression levels of the normal tissue (Table 5). miR-21, miR-146a and miR-182 were more expressed in TNBC than in NTNBC. Indeed, miR-182 exhibited the highest fold change difference with *R* ratio = 4.32 and P value = 0.0001. For miR-21, the *R* ratio = 2.6 and *P* value = 0.01. The miR-146a exhibited *R* ratio = 2.06 and *P* value = 0.01. However, the difference of the mean fold expression of miR-10, miR-17, miR-34 and miR-148a between the two analyzed groups is not significant; *P*>0.05 (Table 5).

**Table 5:** Comparison of the mean fold expression levels (Log 2 transformed) of the different studied microRNAs between both triple negative and non triple negative breast tumors.

Micro RNA	Triple negative breastcancer tumors Foldexpression (mean ± SD)	Non triple negative breastcancer tumors Foldexpression (mean ± SD)	Expressionratio *R*	*P* value
**miR-10a**	0.465±2.27	0.332±1.84	0.24	0.8
**miR-17**	1.64±2.56	0.9±1.06	1.45	0.15
**miR-21**	2.38±2.86	0.86±1.4	2.6	0.01*
**miR-34**	1.44±1.56	2.04±2.01	1.2	0.2
**miR-146a**	2.7193±1.54	1.3834±2.33	2.06	0.01*
**miR-148a**	1.6188±2.54	1.3572±1.21	0.5	0.6
**miR-182**	10.3669±1.64	12.1073±1.77	4.32	0.0001**

Blot letters with *indicate p Value <0.05; Blot letters with **indicate *p* Value <0.01, SD: standard deviation.

### Correlations between the analyzed miRs

In a second step we assessed the correlation between microRNAs fold expression in triple-negative and non triple negative breast cancer using the Spearman Roh method to better understand their interactions (Table 6). Considering the triple negative breast cancer, we observed a group of correlating miRs constituted by miR-10b, miR-21, miR-17, miR-148a and miR-182; while miR-34 and miR-146a appeared to be less correlated with the other studied miRs. The highest correlation was observed between: miR-10b and miR-21 (Spearman’s rho, 0.83; *P*<0.01); miR-10b and miR-17 (Spearman’s rho, 0.82; *P*<0.01); miR-10b and miR-148a (Spearman’s rho, 0.71; *P*<0.01). We also recorded that miR-17 correlates strongly with miR-21; miR-148a and miR-182 (Spearman’s rho, 0.71, 0.74 and 0.6 respectively; *P*<0.01). We observed that miR-21 correlated with both miR-148a and miR-182 (Spearman’s rho, 0.72 and 0.65 respectively; *P*<0.01) along with miR-10a and miR-17. We also noticed a strong correlation between miR-148a and miR-182 (Spearman’s rho, 0.64; p<0.05). Finally we observed that miR-34a correlated weakly with only miR-148a and miR-182 (Spearman’s rho, 0.39 and 0.4 respectively; *P*<0.05) (Table 6).

**Table 6:** Spearman’s rank correlation coefficient between microRNA fold expression levels in triple-negative and non triple negative breast cancer cases.

	miR-10b	miR-17	miR-21	miR-34a	miR-146a	miR-148a	miR-182
**miR-10b**	-	0,825**	0,833**	0,251	-0,049	0,718**	0,475**
**miR-17**	0,143	-	0,717**	0,178	0,169	0,745**	0,608**
**miR-21**	0,154	0,547**	-	0,228	0,084	0,726**	0,650**
**miR-34a**	0,668**	0,205	0,323	-	0,188	0,399*	0,406*
**miR-146a**	0,729**	0,215	0,396*	0,818**	-	0,192	0,309
**miR-148a**	0,127	0,444*	0,381*	0,257	0,338	-	0,643**
**miR-182**	0,166	0,316	0,629**	0,452*	0,505**	0,541**	-

Underlined letters concern the correlation between miRs across triple negative breast cancer. The non underlined letters concern the correlation between miRs across non triple negative breast cancer. Bold letters with *represent *P* value <0.05; Bold letters with **represent *P* value <0.01.

In non triple negative breast cancer, the correlation profile is significantly different. In such tumor, we carried out a strong correlation between miR-34a and miR-146a that was not observed in TNBC (Table 6). On the one hand, the correlation of miR-10b with the other analyzed miRs in NTNBC behaves oppositely compared to that across TNBC and correlates with only miR-34 and miR-146a (Spearman’s rho, 0.66 and 0.72 respectively; *P*<0.01), On the other hand, some correlations appeared with the same strength in both TNBC and NTNBC tumors such as between miR-21 and miR-182, between miR-34a and miR-182 and between miR-148a and miR-182. We also observed that miR-17 correlates strongly with miR-21 and normally with miR-148a (Spearman’s rho, 0.39 and 0.4 respectively; *P*<0.05) and that miR-21 correlates with miR-146a, miR-148a and miR-182 (Spearman’s rho, 0.39, 0.38 and 0.62 respectively; *P*<0.05). miR-34a correlates with both miR-146a and miR-182 (Spearman’s rho, 0.81 and 0.45 respectively; *P*<0.05). We finally observed a correlation between miR-146a and miR-182 (Spearman’s rho, 0.5; *P*<0.05) (Table 6).

### Relationship between miRs expression and clinicopathological features

In a last step we analyzed the relationship between clinicopathological features along with the genico-obstetric history of patients and miRs expression in triple negative and non triple negative mammary tumors. In the triple negative breast cancer group including young patients <38 years old at diagnosis, miR-21 and miR-182 were significantly over-expressed; *P* value = 0.04, while there was no effect of age on expression of any analyzed miR in NTNBC group (Table7). Grade III was associated to miR-10b only in NTNBC (*P* value, 0.01). In TNBC, lymph node metastases occurrence was significantly associated with the expression of miR-10b, miR-21 and miR-182 (*P* value, 0.02; 0.006 and 0.03 respectively); however, it was not associated to any of the analyzed miRs in the other group. The age of the first menstruation >13 years in TNBC was associated to the over-expression of miR-10b; miR-17; miR-21; miR-148a and miR-182 (*P* value, 0.03; 0.006; 0.01; 0.01 and 0.02 respectively) and it was associated only to miR-17 (*P* value, 0.003) in NTNBC group. The age at the first pregnancy >25 years across TNBC was strongly associated to the expression of miR-182 (*P* value, 0.004); however, in relation to NTNBC, it is associated to miR-17 (*P* value 0.03). For women with triple negative breast carcinoma who did not have any completed pregnancy, miR-146a and miR-182 were significantly over-expressed (*P* value, 0.02 and 0.04 respectively). However, in cases of patients with non triple negative breast cancer, we did not record any significant association among the seven studied microRNAs. The non breastfeeding in women with TNBC was significantly associated with only miR-182 (*P* value, 0.02), while in the NTNBC group we did not observe any significant association with any of the studied miRs. The use of the contraceptive pills according to TNBC was strongly associated to all microRNAs except for miR-34 and miR-146a and miR-182 with *P* value <0.05 and in relation to NTNBC, it was significantly associated with the all analyzed microRNAs; *P* value <0.01. The body mass index >30 was significantly associated to miR-21 for both triple negative and non triple negative breast cancer cases (*P* value, 0.03 and 0.04 respectively) (Table7).

**Table 7:** Relationship between clinicopathological features and miRs expression in triple negative and non triple negative mammary tumors.

Clinical features		Triple negative mammary tumors N = 30 *P* value								Non triple negative mammary tumors N = 30 *P* value							
		N/30	miR-10b	miR-17	miR-21	miR-34	miR-146a	miR-148a	miR-182	N/30	miR-10b	miR-17	miR-21	miR-34	miR-146a	miR-148a	miR-182
**Age at diagnoses**	**<38 years**	12	0.12	0.16	0.04	0.43	0.51	0.19	**0.04**	4	0.07	0.95	0.1	0.21	0.43	0.78	0.93
	**>38 years**	18								26							
**Grade**	**II**	3	0.1	0.26	0.2	0.5	0.17	0.36	0.43	9	**0.01**	0.22	0.72	0.08	0.21	0.51	0.72
	**III**	27								19							
**Stage**	**T1–T2**	19	0.09	0.55	0.11	0.72	0.98	0.42	0.41	23	0.28	0.53	0.82	0.61	0.83	0.76	0.72
	**T3–T4**	11								5							
**LNM**	**No**	20	**0.02**	0.12	**0.006**	0.18	0.57	0.09	**0.03**	24	0.18	0.65	0.84	0.55	0.84	0.76	0.89
	**Yes**	10								06							
**Distant metastases**	**No**	23	0.24	0.3	0.54	0.57	0.41	0.32	0.62	26	0.23	0.58	0.63	0.43	0.73	0.95	0.73
	**Yes**	07								04							
**AFM**	**<13 years**	11	**0.03**	**0.006**	**0.01**	0.6	0.44	**0.01**	**0.02**	09	0.37	**0.0001**	0.1	0.5	0.09	0.58	0.65
	**>13 years**	13								14							
	**Unknown**	06								07							
**AFP**	**No pregnancy**	06	0.24	0.43	0.07	0.16	0.2	0.12	**0.004**	10	0.3	**0.03**	0.42	0.06	0.13	0.9	0.49
	**<25 years**	10								08							
	**>25 years**	09								09							
	**Unknown**	05								03							
**NCP**	**0**	10	0.48	0.25	0.2	0.26	**0.02**	0.23	**0.04**	14	0.79	0.56	0.28	0.47	0.67	0.46	0.98
	**one at least**	15								09							
	**Unknown**	05								07							
**Breastfeeding**	**No**	14	0.85	0.31	0.15	0.56	0.12	0.17	**0.02**	14	0.9	0.65	0.43	0.86	0.89	0.57	0.58
	**Yes**	12								10							
	*Unknown	04								06							
**Contraceptive pills**	**No**	16	**0.001**	**0.009**	**0.003**	0.93	0.65	**0.03**	0.08	19	**0.03**	**0.009**	**0.003**	**0.008**	**0.01**	**0.05**	**0.05**
	**Yes**	10								06							
	**Unknown**	05								05							
**BM**	**<30**	13	0.15	0.3	**0.03**	0.28	0.69	0.6	0.52	09	0.35	0.25	**0.04**	0.87	0.72	0.72	0.95
	**>30**	10								18							
	**Unknown**	07								03							

*P* value in blot ≤0.05; BMI: Body Mass Index; NCP: Number of Completed Pregnancy; AFP: Age of First Pregnancy; AFM: Age of First Menstruation; LNM: Lymph Node Metastases.

According to our results, we consider miR-10b, miR-21 and miR-182 as potential biomarkers that could play a predictive role in the lymph node metastases occurrence across triple negative breast cancer and miR-10b as biomarker that could predict the occurrence of high grade III in the non triple negative breast cancer group. Moreover, we assessed the miRs distribution in the two groups, where we showed a clear difference in the distribution of these three microRNAs across the two analyzed groups. Indeed, miR-10b, miR-21 and miR-182 in TNBC are significantly over-expressed in patients with lymph node metastases (*P* value, 0.02, 0.006 and 0.03 respectively). However, the distribution of these microRNAs within non triple negative breast carcinoma among patients with and without lymph node metastases were not significant (*P* value <0.05) (Figure S1).

Then, we scored the expression of miR-10b, miR-12, and miR-182 by curve ROC to confirm their association to lymph node metastases across TNBC and to Grade III according to non triple negative breast cancer by measuring their degree of sensibility and specificity with respect to this type of cancer (Figure S2) and (Figure S3). miR-21 could be a good biomarker for the occurrence of lymph node metastases within the triple negative breast cancer from a cut-off value of 2.9004 with a sensitivity of 80% and a specificity of 85% and an AUC value of 0.82. In addition, miR-10b and miR-182 were significantly over-expressed in mammary tumors with TNBC to predict the occurrence of lymph node metastases from a cut-off value of 0.8732 and 3.2992, respectively, with a sensitivity of 70% and 80% respectively, and a specificity of 80% for both miRs (Figure S2).

Moreover, it appears well that miR10b could be considered as a biomarker for the occurrence of a high grade in non triple negative breast cancer from a cut-off value of −0.4424 with a sensitivity of 78% and a specificity of 72% and an AUC value of 0.78 (Figure S3). Nevertheless, we considered the additive effect of hormonal factors (age of first menstruation, age at the first pregnancy, use of the contraceptive pills, breastfeeding) that have been associated with miR deregulation in TNBC. Taking into account two, three or four hormonal risk factors, we constituted subgroups across triple negative breast patients and compared miRs fold expression (mean ± SD) in tumors according to the presence or absence of the additive effect of these hormonal features (Table 8). Except for miR-34a and miR-146a, we found that all the other micro-RNAs were significantly associated with TNBC group presenting at least two hormonal risk factors. These results are in agreement with our previous findings. miR-10b which behaved as non associated with breast tumor when the patients were not stratified, is confirmed to be associated with the additive hormonal effect with *P* value = 0.025 and expression ratio *R* = 0.32 (Table 8).

**Table 8:** Comparison of miRs fold expression levels according to 0 or 1 hormonal risk factor and to the hormonal additive effect (age of first menstruation, age at the first pregnancy, use of the contraceptive pills and breastfeeding) among triple negative breast patients.

	0 or 1 hormonal risk factor	2,3 or 4 hormonal risk factors		
miR	Fold expression (mean ± SD)	Fold expression (mean ± SD)	P value	Expression ratio *R*
**miR-10**	–0.6980±1.898	1.1394±2.238	**0.025**	0.32
**miR-17**	0.2037±1.478	2.4758±2.722	**0.006**	2.36
**miR-21**	0.5288±1.860	3.4542±2.819	**0.002**	2.21
**miR-34a**	1.3640±1.270	1.4894±1.747	0.82	0.79
**miR-146a**	2.4741±1.386	2.8612±1.652	0.49	0.24
**miR-148a**	–0.0585±1.641	2.5899±2.498	**0.002**	2.03
**miR-182**	1.9118±1.329	4.2027±2.705	**0.004**	3.73

*P* value in blot <0.05, SD: standard deviation.

## General Discussion

The involvement of miRNAs as regulators of gene expression identifies them as a potential candidates of diagnostic and prognostic indicators and therapeutic targets [18]. Therefore, miRs expression profile could predict prognosis, support diagnosis, and improve therapy responses in cancer [18]. Extensive research in the last decade has implicated miRNAs as master regulators of cellular processes with essential role in cancer initiation, progression, and metastasis, making them promising therapeutic tools for cancer management [19]. Indeed, miRs signatures could help on the classification of human cancer of unknown primary origin as well as poorly differentiated tumors [20]. Hence, the microRNA profiles could potentially be used to develop predictive biomarkers in cancer [21].

We explored here the expression levels of seven miRNAs in triple negative and non triple negative breast cancer and compared them to the corresponding levels of their adjacent normal tissues. The studied microRNAs were selected according to the literature, as shown in Table 1, taking into account their essential role in cell growth and angiogenesis [22].

First, we showed that all the analyzed microRNAs except for miR-10b were over-expressed in tumor tissues than in normal ones in both triple negative and non triple negative breast cancer.

These results are in agreement with other studies of the same microRNAs, which are involved in angiogenesis and cell proliferation, in several cancer types [23].

Second, comparing the fold expression between the two groups of TNBC and NTNBC, we also showed that the deregulation is significantly different between the two analyzed groups for miR-21, miR-146a and miR-182. Third, the correlation profile within miRs expression level is different between TNBC and NTNBC specifically in relation to miR-10b, miR-34a and miR-146a. Fourth, the expression level of all the studied miRs (except for miR-34a in TNBC) is associated with at least one feature related to hormonal status, in both groups, indicating that this status could be determinant as an important risk factor leading to breast cancer. Last, the expression of miR-10b and miR-21 are correlated to bad clinical presentation particularly to lymph node metastasis in TNBC and grade III for miR-10b in NTNBC.

In the present study, miR-10b seemed not to be over-expressed in mammary tumors; in NTNBC group it was associated with the occurrence of a high grade (III) and correlates with both miR-34 and miR-182. This correlation enhances angiogenic activity of endothelial cells in hepatocellular carcinoma [24], and has been described in the high grade of prostate cancer [25]. However, in TNBC group, miR-10b was associated to lymph node metastases and correlates with miR-17, miR-21, and miR-148a. These interactions were well known for promoting cell growth, angiogenesis and proliferation in various tumor types [26]. In addition, miR-10b has been described as strongly involved in cell motility metastases and invasion [27], [28], but its involvement in breast cancer subtypes is not well documented. The association to lymph node metastases occurrence could be explained by the selection of a tumor phenotype characterized by the expression of genes involved in motility and invasion probably in relation with miR-10b expression that could be under influence of environmental features and to the life style of patients.

In this paper, the impact of tumor micro-environment on miR expression was demonstrated particularly with miR-21 which is influenced by adipose tissue since we showed an important association of miR-21 with the BMI “Body Mass Index” for both triple negative and non triple negative breast cancer. According to the literature, miR-221, that correlates with miR-21, is strongly involved in adipose cells and consequently leads to breast cancer [29], [30]. In addition, miR-21′s association with miR-148a and miR-182 in both TNBC and NTNBC according to Spearman’s rank correlation coefficient has been found to be involved in different tumors, including glioblastoma, lung, stomach, pancreatic, colon, prostate and breast, [31] as well as in invasive cancer cells and tumor metastases [31].

In TNBC group, the expression of miR-21 is related to hormonal status particularly to the age of first menstruation and to the use of the contraceptive pills and it strongly correlates with almost all the studied miRs except for miR-34 and miR-146a. This correlation was described to promote cell proliferation and is often involved in several carcinogenesis mechanisms in breast cancer [32].

In fact, it was described that miR-21 is involved in positive hormonal breast carcinoma especially in those expressing HER2 that upregulate miR-21 and, therefore, it is involved in positive hormonal breast cancer subtypes since it appeared to interact with the α ER pathway [33], [34], [35]. However, according to our results, we found that this microRNA is more expressed in the negative hormonal receptors among the Tunisian population. Indeed, it may be due to the combination of the lifestyle and the patients’ history which could vary among populations.

Concerning miR-146a, our results prove that it was over-expressed in tumor tissue as compared to normal one in both analyzed groups of TNBC and NTNBC, but it seemed to be more involved in triple negative breast cancer group. Our findings are in agreement with other studies; indeed, miR-146a has been described to target the 3′UTR of *BRCA1* gene that is altered in almost triple negative breast cancer cases (25%) [36], [37]. Several studies focused on miR-146a as an interesting biomarker for an early cancer diagnosis and as a potential target for gene therapy in cancer. It was also shown that polymorphisms in miR-146a were associated to cancer risk [38]. The role of miR-146a in the regulation of inflammation induced via the innate immune response is largely documented [39]. It was identified that miR-21 and miR-146a were detected in the circulating serum of patients’ breast cancer [40]. According to our results, miR-146a seemed to act independently of the other analyzed miRs in TNBC group. Consequently, we did not record any significant Spearman Roh correlations between miR-146a and the others. This supports the idea that this miR could behave specifically in triple negative breast carcinoma subtype and could act alone independently of other micro-RNAs since it targets *BRCA1* gene.

In the case of miR-182, which has been described to be upregulated in breast carcinoma, to target specifically *BRCA1* gene and to be involved in the resistance to *PARP-1* inhibitors [41], [42], we identified a relationship between miR-182 expression and clinical outcome. Interestingly, an association between miR-21 and miR-182 expression to young patients with triple negative breast cancer, was proved in this work. This observation supports our clinical findings concerning the age at diagnosis where young women were significantly higher in TNBC than in NTNBC group. In addition, these two microRNAs correlated strongly together in both analyzed groups along with miR-10b in TNBC. These correlations could explain in part the involvement of the three miRs (miR-10b, miR-21 and miR-182) in the occurrence of the lymph node metastases within triple negative breast cancer group.

According to our findings, the association of microRNAs with genico-obstetric history of patients such as the age of first menstruation upper 13 years old was observed in TNBC for all microRNAs except for miR-34a and miR-146a whereas in NTNBC this feature is associated just with miR-17. We also observed that the age of first pregnancy upper than 25 years old is significantly associated to miR-182 and miR-17 respectively in TNBC and NTNBC. This observation is in agreement with other studies showing that the young age of first pregnancy under 25 years old is a measure of protection against breast cancer [43], [44]. In addition, women with no completed pregnancy and who have never breastfed had significant association with the expression of miR146a and miR-182 in TNBC. These two microRNAs correlate strongly together according to Spearman’s rank correlation coefficient in NTNBC. miR146a and miR-182 combined together along with miR-148a, were found to play a role in cell growth, angiogenesis, proliferation and invasion, through the silencing of the tumor suppressors tropomyosin-1 (TPM1) and programmed cell death gene-4 (PDCD4) [45]. The correlation between miR-182 and miR-148a has already been described to be involved in medulloblastoma and metastases mechanisms [46].

We also showed that the use of the contraceptive pills was strongly associated to all the analyzed microRNAs expression in TNBC except for miR-34a and miR-146a, while it is associated to all microRNAs in NTNBC, suggesting the important role assigned to oral contraceptive to the onset of breast cancer. This observation is in agreement with other works reporting the increased risk of breast cancer among women who consume oral contraceptive [47], [48]. Our observation suggests that the hormonal status and the genico-obstetric history of patients are important to direct miRs expression as potential prognostic biomarkers to predict some clinical features. In non triple negative breast cancer, the association of all the miRs studied with the use of the contraceptive pills reveals the importance of hormonal factors in miR deregulation. In non triple negative breast tumors, the additive hormonal effect seems to have an important role to direct the oncomiR expression. In addition, despite the presence of negative hormonal receptors in these tumors, the additive hormonal effect seems to play an important role to improve the oncomiR expression in triple negative breast cancer.

According to our findings, miR-10b, miR21 and miR-182 could be considered as diagnostic and prognostic biomarkers to predict lymph node metastases occurrence, this observation was supported by the Roc curve that predicts with a good specificity and sensibility the involvement of these microRNAs in the occurrence of lymph node metastases. In addition, the three microRNAs significantly correlate together according to Spearman method in TNBC, whereas we did not record a significant correlation among the same microRNAs in NTNBC. Further studies should be realized to better confirm their implication in triple negative breast cancer.

## Conclusion

The direction of microRNAs expression is strongly influenced by environmental factors and the genico-obstetric history of patients. In our case, miR-10b, miR-21 and miR-182 could be defined as biomarkers to predict both lymph node metastases in triple negative breast cancer and miR-10b to predict grade III occurrence in non triple negative breast cancer.

## Supporting Information

Figure S1 **Distribution of miR-10b, miR-21 and miR-182 according to lymph node metastases status in triple negative and non triple negative breast cancer.**
(DOC)

Figure S2 **Roc curve of the lymph node metastases occurrence prediction according to miR-10b, miR-21, miR-148a and miR-182 fold expression among triple negative breast cancer cases.**
(DOC)

Figure S3 **Roc curve of the high grade (III) occurrence prediction according to miR-10b fold expression among non triple negative breast cancer cases.**
(DOC)
