# Supplementary material for: MicroRNAs Expression in Triple Negative vs Non Triple Negative Breast Cancer in Tunisia: Interaction with Clinical Outcome
Source: PLoS One. 2014 Nov 4;9(11):e111877. doi: 10.1371/journal.pone.0111877 (PMC4219794; doi:10.1371/journal.pone.0111877)

**Figure S1:** Distribution of miR-10b, miR-21 and miR-182 according to lymph node metastases status in triple negative and non triple negative breast cancer.


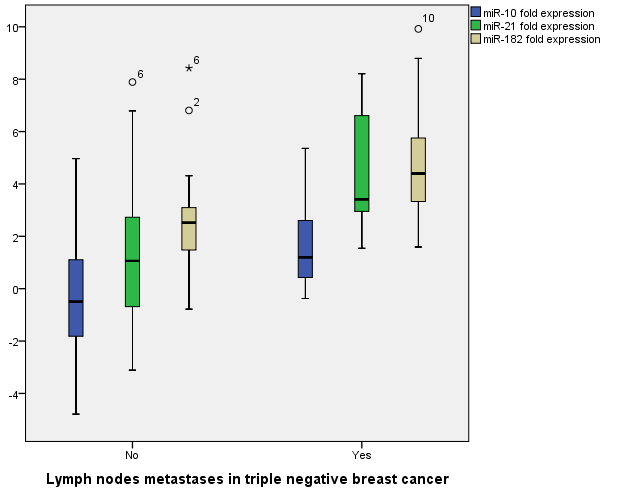

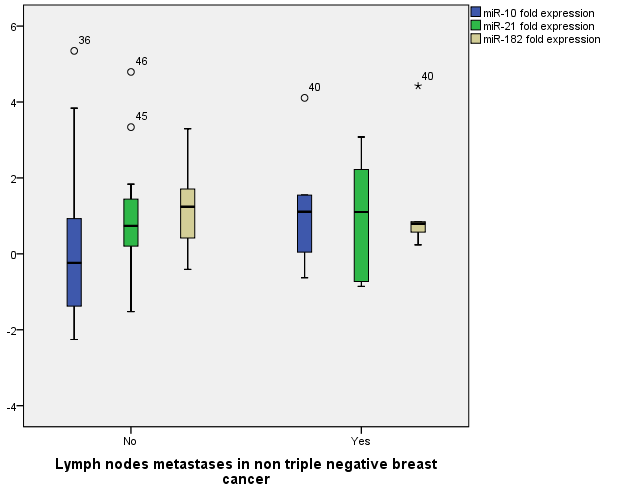

Supplement: Figure S1 — Distribution of miR-10b, miR-21 and miR-182 according to lymph node metastases status in triple negative and non triple negative breast cancer. (DOC) [file pone.0111877.s001.doc]
