# Supplementary material for: MicroRNAs Expression in Triple Negative vs Non Triple Negative Breast Cancer in Tunisia: Interaction with Clinical Outcome
Source: PLoS One. 2014 Nov 4;9(11):e111877. doi: 10.1371/journal.pone.0111877 (PMC4219794; doi:10.1371/journal.pone.0111877)
Supplement: Figure S3 — Roc curve of the high grade (III) occurrence prediction according to miR-10b fold expression among non triple negative breast cancer cases. (DOC) [file pone.0111877.s003.doc]

**Figure S3:** Roc curve of the high grade (III) occurrence prediction according to miR-10b fold expression among non triple negative breast cancer cases.

**Roc Curve**


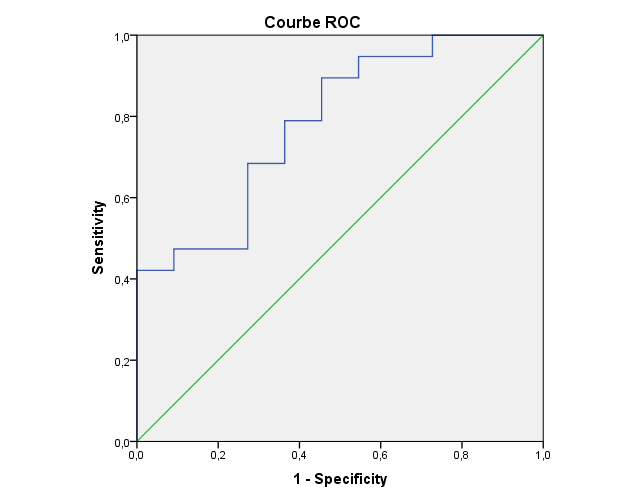

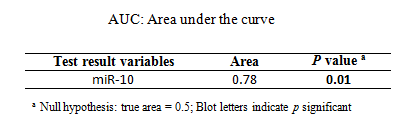


**Roc Curve**
